# Serum sCD40L and galectin-3 in the prognosis prediction of patients with acute ischemic stroke

**DOI:** 10.5937/jomb0-61699

**Published:** 2026-05-22

**Authors:** Zhiqin Chen, Quanhua Xiao, Kunqing Luo, Lin Shuping, Fan Zhaoyang, Xianliang Lai

**Affiliations:** 1 Nanchang University, Jiangxi Medical College, The Second Affiliated Hospital, Department of Neurosurgery, Nanchang City, China; 2 Qilu Hospital of Shandong University, Neurology Department, Jinan City, China

**Keywords:** serum soluble sCD40 ligand, serum galectin-3, acute ischemic stroke, therapeutic effect, prognosis and outcome, predictive value, serumski rastvorljivi sCD40 ligand, serumski galektin-3, akutni ishemijski moždani udar, terapijski efekat, prognoza i ishod, prognostička vrednost

## Abstract

**Background:**

To explore the value of serum soluble CD40ligand (sCD40L) and galectin-3 (galectin-3) for predictingthe therapeutic effect and prognosis of patients with acuteischemic stroke (AIS).

**Methods:**

180 AIS patients admitted to the hospitalbetween March 2023 and March 2025 were selected asresearch participants. All patients received emergencytreatment with tenecteplase (TNK-tPA) combined withXuesaitong. Based on the therapeutic effect, patients weredivided into two groups: an effective group and an ineffective group. Serum sCD40L and galectin-3 levels weremeasured in both groups before treatment and 4 weeksafter, and the levels were compared. After discharge, allpatients were monitored for 3 months, and, based on theirmodified Rankin Scale (mRS) score, those with a goodprognosis and those with a poor prognosis were assignedto two groups. Using multivariate logistic regression, therisk factors associated with poor prognosis and inadequatetreatment in AIS patients were examined. Serum levels ofsCD40L and galectin-3 were analysed using a receiveroperating characteristic (ROC) curve to assess their prognostic value for poor prognosis and ineffective treatment inAIS patients.

**Results:**

Of the 180 research subjects, 146 were effectivelytreated (effective group), while 34 were ineffective (ineffective group). There were 142 patients with a good prognosisand 38 with a poor prognosis, representing an incidence of21.11%. After 4 weeks of treatment, serum levels ofsCD40L and galectin-3 were lower in the successful group than in the unsuccessful group (P&lt; 0.05). Serum levels ofsCD40L and galectin-3 were higher in the poor-prognosisgroup than in the favourable-prognosis group (P&lt; 0.05).Multivariate logistic regression analysis showed that AISpatients with elevated serum galectin-3 and sCD40L levelswere at increased risk of inadequate treatment and pooroutcomes (P&lt; 0.05). The areas under the curve (AUCs) forserum galectin-3 and sCD40L in predicting treatment failure in AIS patients were 0.665 and 0.691, respectively,according to ROC analysis; the specificities were 70.08%and 77.51% , respectively, and the sensitivities were 62.53%and 62.58%, respectively. The combined prediction modelfor ineffective treatment using serum galectin-3 andsCD40L yielded an AUC of 0.784, with specificities andsensitivities of 65.18% and 81.25%, respectively. For predicting poor prognosis, serum galectin-3 and scd40ldemonstrated AUCs of 0.774 and 0.838, respectively; theirspecificities were 67.58% and 75.36% , respectively, whiletheir sensitivities were 75.06% and 80.04% , respectively.The combined prediction of serum galectin-3 and scd40lfor poor prognosis was less effective, with an AUC of 0.919,a specificity of 60.05%, and a sensitivity of 90.63%.

**Conclusions:**

Both sCD40L and serum galectin-3 levelshave some prognostic value for poor prognosis and ineffective treatment in AIS patients, and combining their detection can significantly improve predictive efficacy.

## Abstract (sr)

**Uvod:**

Cilj je bio ispitivanje vrednosti serumskog rastvorljivog CD40 liganda (sCD40L) i galektina-3 (galectin-3) upredviđanju terapijskog efekta i prognoze kod pacijenata saakutnim ishemijskim moždanim udarom (AIM).

**Metode:**

U istraživanje je uključeno 180 pacijenata sa AIMkoji su bili hospitalizovani između marta 2023. i marta2025. godine. Svi pacijenti su primili hitnu terapiju tenekteplazom (TNK-tPA) u kombinaciji sa preparatomSjuesejtong (Xuesaitong). Na osnovu terapijskog efekta,pacijenti su podeljeni u dve grupe: efektivnu i neefektivnu.Nivoi sCD40L i galektina-3 u serumu su mereni kod obegrupe pre početka terapije i četiri nedelje nakon nje, azatim su upoređeni. Nakon otpusta, svi pacijenti su pracenitokom tri meseca, i prema modifikovanoj Rankinovoj skali(mRS) podeljeni su na grupu sa dobrom i grupu sa losomprognozom. Visestrukom logističkom regresionom analizom ispitani su faktori rizika povezani sa losom prognozomi neadekvatnim terapijskim odgovorom kod pacijenata saAIM. Analizom ROC krive je procenjena prognostička vrednost serumskih nivoa sCD40L i galektina-3 za losu prognozu i neefikasno lečenje.

**Rezultati:**

Od 180 ispitanika, 146 je imalo efektivan terapijski odgovor (efektivna grupa), a 34 nisu (neefektivnagrupa). Ukupno 142 pacijenta su imala dobru prognozu,dok je 38 imalo losu prognozu, sto predstavlja incidenculose prognoze od 21,11% . Nakon četiri nedelje terapije,nivoi sCD40L i galektina-3 u serumu bili su niži u uspesnolečenoj grupi u odnosu na neuspesno lečenu (P&lt; 0,05).Nivoi sCD40L i galektina-3 bili su visi u grupi sa losom prognozom u poređenju sa grupom sa povoljnom prognozom (P&lt; 0,05). Visestruka logistička regresiona analizaje pokazala da pacijenti sa AIM i visokim nivoima galektina-3 i sCD40L u serumu imaj'u povećan rizik od neadekvatnog terapijskog odgovora i loseg ishoda (P&lt; 0,05).ROC analiza je pokazala da su povrsine ispod krive (AUC)za galektin-3 i sCD40L u predviđanju neefikasnog lečenjabile 0,665 i 0,691; odgovarajuće specifičnosti su bile70,08% i 77,51% , a senzitivnosti 62,53% i 62,58%.Kombinovana predikcija neefikasnog lečenja korišćenjemoba biomarkera dala je AUC od 0,784, sa specifičnošćuod 65,18% i senzitivnošću od 81,25% . Za predviđanjelose prognoze AUC vrednosti galektina-3 i sCD 40Liznosile su 0,774 i 0,838; specifičnosti 67,58% i 75,36% ,a senzitivnosti 75,06% i 80,04% . Kombinovana predikcijaserumskih galektina-3 i sCD40L za losu prognozu pokazala je AUC od 0,919, sa specifičnosću od 60,05% i senzitivnosću od 90,63%.

**Zaključak:**

Nivoi serumskog sCD40L i galektina-3 imajuodređenu prognostičku vrednost u predviđanju lose prognoze i neefikasnog lečenja kod pacijenata sa AIM, a njihova kombinovana detekcija može značajno da poboljsatačnost predviđanja.

## Introduction

Acute ischemic stroke (AIS) is a common cerebrovascular disease with sudden onset and rapid progression [1][2][3]. It is mainly caused by the narrowing of blood vessels in the brain, which restricts blood flow. At present, in clinical practice, methods such as eliminating oxygen-free radicals, improving cerebral circulation, nourishing nerves and antioxidation are widely adopted to alleviate patients' clinical symptoms and delay the continuous deterioration of the disease. Previous studies [4][5][6] have evaluated the therapeutic effect in mostly AIS patients depending on variations in the score on the National Institutes of Health Stroke Scale (NIHSS). However, this method has obvious drawbacks, including significant subjectivity and poor repeatability. Previous studies [7][8][9] have confirmed that serum markers have high diagnostic value for assessing the clinical efficacy and prognosis of various cardiovascular diseases. The development of AIS disease and the decline in neurological function are directly linked to large and medium-sized arterial lesions, which are mainly caused by atherosclerosis, according to studies [10][11][12]. Galectin-3 has been proven to have high clinical value in evaluating the stability and size of atherosclerotic plaques, and it is also correlated with the number of diseased blood vessels. Soluble CD40 ligand (sCD40L) is also associated with arterial thrombosis and atherosclerosis [13].

One of the leading causes of mortality and adult impairment in the globe is acute ischemic stroke (AIS) [14]. Timely prognosis assessment is crucial for guiding individualised clinical treatment, optimising resource allocation, implementing early rehabilitation interventions, and enhancing long-term functional outcomes [15]. However, these methods still have limitations in terms of predictive sensitivity and specificity, as well as in their ability to reveal potential pathophysiological mechanisms. Therefore, exploring new serum biomarkers that can more accurately, objectively and comprehensively reflect the pathological process and prognosis of AIS patients has always been the focus of stroke research. sCD40L (soluble CD40 ligand), a key mediator of platelet activation and the inflammatory response, plays a significant role in the occurrence and development of AIS. Its elevated level is closely associated with thrombosis, amplification of the inflammatory cascade, endothelial dysfunction, and plaque instability. Galectin-3 (galectin-3) is an essential β-galactosidase-binding protein that participates in regulating various pathological processes, such as the inflammatory response, myocardial fibrosis, and tissue remodelling [16][17][18]. Its prognostic value in cardiovascular diseases has received attention, but its role in nerve injury repair and in predicting adverse outcomes after AIS still needs to be further explored [19].

An evaluation of the associations of these two novel biomarkers with clinical outcomes, such as the severity of neurological deficits in patients, short-term and medium-term functional recovery (such as modified Rankin scale scores), and recurrence risk, is expected to provide a new perspective for understanding complex thrombotic inflammation and neural repair mechanisms after AIS. More importantly, if the two have independent or collaborative prognostic predictive capabilities, they may provide clinicians with earlier, more sensitive, and more reliable prognostic assessment tools, ultimately optimising the individualised prognostic assessment and management of AIS patients. Improve their quality of life and long-term outcomes.

## Materials and methods

### General information

One hundred eighty AIS patients admitted to our hospital between March 2023 and March 2025, 95 men and 85 women, were selected as research participants. With an average age of 68.01±8.05 years, the ages varied from 61 to 88. With an average of 22.48±2.14 kg/m^2^, the body mass index ranged from 19.94 to 25.45 kg/m^2^.

Underlying diseases: Seventy-three patients had hypertension, 65 patients had diabetes, and 92 patients had hyperlipidemia.

Our institution's Medical Ethics Committee approved the study, and each participant freely completed the informed consent form (HKYS-2025-A0211).

### Inclusion criteria and exclusion criteria

Inclusion criteria: (1) clearly diagnosed with AIS through imaging examinations such as CT and MRI, with the diagnostic criteria; (2) aged >60 years; (3) first onset of the disease, the first treatment received, and the time from onset to admission was 4 hours; and (4) complete clinical data. Exclusion criteria: (1) had combined liver or kidney dysfunction; (2) had concurrent malignant tumours; (3) had concurrent acute or chronic infections before enrollment; and (4) had poor compliance and inability to cooperate in completing this research; (5) Those with concurrent immune or hematological diseases; (6) those who have participated with other clinical researchers simultaneously.

### Treatment methods

After admission, all patients received conventional treatments, including traditional oxygen inhalation, mannitol to reduce intracranial pressure, nutritional support, and maintenance of body temperature and water-electrolyte balance. On this basis, 0.9 mg/kg tinalplase (TNK-tPA) was combined with 200 mg Xuesaitong (Yunnan Baiyao Group Co., Ltd., National Drug Approval No. Z53021499, specification: For emergency treatment (2 mL 100 mg), Within one minute, 10.0% of the total dose of TNK-tPA, which had a maximum dose of 90 mg, was administered intravenously. The remaining 90.0% dose was subsequently dissolved in 250 mL of 0.9% sodium chloride solution and intravenously infused within 1 hour. Xuesaitong is administered via intravenous infusion once daily. The therapeutic effect was evaluated after 4 weeks of continuous treatment.

### Detection of serum sCD40L and galectin-3 levels

Five mL of fasting elbow venous blood was collected from all enrolled patients in the early morning of the day following admission. Samples were collected using BD Vacutainer® (REF 367983). After standing at room temperature for 30 minutes, immediately Centrifuge at 3000 g and 4°C for 15 minutes (Centrifuge 5804 R, Eppendorf). After serum separation, aliquot 200 μL into sterile 0.5 mL cryotubes (Axygen, PCR-05-C) and transfer to a -80°C ultra-low-temperature refrigerator (Thermo Scientific ULT1586) for light-protected storage for up to 2 hours until detection. Serum sCD40L and galectin-3 concentrations were measured using a double-antibody sandwich enzyme-linked immunosorbent assay (ELISA), performed strictly according to the kit instructions. sCD40L uses the Human sCD40L Quantikine ELISA Kit (R&D Systems, catalogue Number SCD40) Galectin-3 employs the Human Galectin-3 ELISA Kit (Abcam, ab279416). Before the test, place the frozen serum in a 4 refrigerator to thaw slowly. Avoid repeated freezing and thawing ( 2 times). Each batch of experiments included standard substances (concentration range: sCD40L 0.16-10.00 ng/mL, galectin-3 0.1-32 ng/mL) and quality control serum (R&D Systems, QC01). Both the samples and the standards were subjected to double-well detection. The absorbance was measured at 450 nm using a microplate reader (BioTek Synergy H1), and the concentration was calculated from a standard curve (four-parameter logistic regression). The intrabatch coefficient of variation (CV) was all controlled at less than 8%, and the inter-batch CV was less than 10%.

### Laboratory testing reagents and equipment

In this study, commercial enzyme-linked immunosorbent assay (ELISA) kits were used to detect serum markers: The sCD40L detection uses the R&D Systems Human sCD40L Quantikine ELISA Kit (item No. SCD40, specification 96T), which contains 96-well plates (polystyrene strips) pre-coated with anti-human CD40L monoclonal antibody. (Item No. SCD401), biotinylation detection antibody (Item No. SCD402) and streptavidin-HRP (Item No. SCD403); galectin-3 detection was carried out using the Abcam Human Galectin-3 ELISA Kit (Item No. ab279416, specification 96T), which contained microplates (Item No. Ab279416-1) pre-coated with anti-human galectin-3 antibody (clone No. 9H3.1). Sample processing was performed using BD Vacutainer® SST™ (catalogue number 367983), and centrifugation was carried out using an Eppendorf Centrifuge 5804 R (rotor number A-4-62) at 3000 g at 4°C for 15 minutes. The micropipettes are of the Eppendorf Research® plus series (volume range 0.5-10 μL, item No. 3123000020; 10-100 pL, item No. 3l23000044). The microplate reader uses the BioTek Synergy H1 multifunctional detection system (model SH1SA, equipped with Gen5 3.08 software), and the plate washing uses the Thermo Scientific Wellwash Versa microplate washer (item number 51171870). The cryopreservation tubes were Axygen pyrogen-free PCR tubes (catalogue number PCR-05-C), and the samples were stored in a Thermo Scientific ULT-1586 ultra-low-temperature freezer (-80°C ±3°C, temperature recorder model TR-100).

### Therapeutic effect evaluation

The therapeutic effect evaluation criteria were as follows: After 4 weeks of treatment, an NIHSS score reduction of more than 90.0% was considered cured, a reduction of more than 46.0% to 90.0% was considered markedly effective, a reduction of 18.0% to 46.0% was considered effective, and a reduction of less than 18.0% was considered ineffective.

### Follow-up investigation

After the patient is discharged from the hospital, regular follow-ups are conducted by phone, WeChat, and other means. Medication guidance is provided to the patient, and they are reminded to return to the hospital for re-examination in time if they feel unwell. The follow-up period was three months. The patients' clinical prognosis was assessed using the modified Rankin Scale (mRS): 0 points were assigned for asymptomatic or imperceptible symptoms. The presence of symptoms without obvious disability was scored as 1 point. Mild disability but still able to take care of oneself in daily life is rated as 2 points. People with an mRS score greater than 2 were in the poor-prognosis group, and those with an mRS score less than 2 were in the favourable-prognosis group.

### Statistical methods

Data analysis was conducted using SPSS 19.0. The χ^2^ test was used to compare groups, and count data are presented as percentages or counts. x̄±s is the expression for the measurement data that follows a normal distribution. Multivariate logistic regression was used to analyse risk factors associated with ineffective treatment and poor prognosis in AIS patients. The predictive effectiveness of blood galectin-3 and sCD40L levels for ineffective treatment and poor prognosis in individuals with AIS was examined using receiver operating characteristic (ROC) curves.

## Results

### Therapeutic effects

All patients in this study completed follow-up, with no dropouts or loss to follow-up during the study period. Of them, 34 patients were classified as unsuccessful since their NIHSS score drop was less than 18%. The effective group comprised the remaining 146 patients.

The overall therapeutic effect showed that 146 cases (81.11%) were effective and 34 were ineffective. After 4 weeks of treatment, the levels of serum sCD40L and galectin-3 in the effective group decreased significantly from baseline, and both were significantly lower than those in the ineffective group (P<0.05), suggesting that remission of inflammation and fibrosis-related pathways was consistent with the clinical improvement. When ROC analysis was used to identify the poor therapeutic effect, the AUCs for galectin-3 and sCD40L were 0.665 and 0.691, respectively, corresponding to specificities of 70.08% and 77.51%, and sensitivities of 62.53% and 62.58%, respectively. Combining the two increased the AUC to 0.784, the sensitivity to 81.25%, and the specificity to 65.18%, which was significantly better than that of a single indicator.

### Galectin-3 and sCD40L levels in the serum of the ineffective and effective groups before and after treatment

Serum galectin-3 and sCD40L levels did not differ significantly between the two groups before therapy (P>0.05). Serum levels of sCD40L and galectin-3 were lower in the effective group than in the ineffective group following 4 weeks of treatment (P<0.05) (Table 1).

**Table 1:** Comparison of serum galectin-3 and sCD40L levels before and after treatment between ineffective and effective groups.

Group	n	Galectin-3 (ng/mL)		sCD40L (pg/L)	
		Before treatment	After 4 weeks <br>of treatment	Before treatment	After 4 weeks <br>of treatment
ineffective group	34	13.29±4.53	10.28±3.42	363.42±60.27	358.23±64.13
effective group	146	12.95±5.14	7.57±2.73	362,89±61.03	273.84±20.37
t		0.384	4.312	0.046	7.584
P		0.702	<0.001	0.963	<0.001

Serum sCD40L and galectin-3 levels in the two groups were measured before treatment and 4 weeks after treatment, and compared. The results showed that four weeks after treatment, the levels of the two indicators in the effective group were significantly lower than those in the ineffective group (P<0.05), while the ineffective group still maintained a relatively high level at the same time point. Based on longitudinal changes at baseline and follow-up time points, the greater the therapeutic effect, the more pronounced the downward trend in the two indicators; conversely, the decline is limited or the levels remain persistently high.

sCD40L and galectin-3 can reflect the biological response to TNK-TPA combined with Xuesaitong treatment. Their absolute levels at 4 weeks after treatment show good discriminatory ability to distinguish between effective and ineffective, consistent with the direction of clinical efficacy evaluation.

### Multivariate logistic analysis of factors affecting the ineffectiveness of treatment in AIS patients

The serum galectin-3 and sCD40L levels of the two patient groups after 4 weeks of treatment were used as independent variables (all entered as original values), and the clinical effect of the patients after treatment was used as the dependent variable (assignment: ineffective = 1, effective = 0). A multivariate logistic regression analysis was carried out. Elevated serum levels of sCD40L and galectin-3 were found to be risk factors for AIS patients' unsuccessful treatment (P<0.05) (Table 2).

**Table 2:** The influence of serum galectin-3 and sCD40L levels on AIS patients' multivariate logistic regression analysis of treatment efficacy.

Factor	β	SE	Wald χ^2^	P	OR (95%CI)
Constant	-12.031	2.827	18.111	-	-
Galectin-3	0.318	0.076	17.508	<0.001	1.374 (1.184~1.595)
sCD40L	0.285	0.05	30.039	<0.001	1.330 (1.201~1.472)

Multivariate logistic regression was used to analyse the factors associated with poor therapeutic effect. After correcting for common baseline characteristics as covariates, elevated levels of serum sCD40L and galectin-3 were significantly associated with treatment ineffectiveness and were independent risk factors (all P<0.05), showing a positive correlation. That is, the higher the index level, the greater the risk of poor therapeutic effect. The above results are consistent with the moderate improvement in single- and combined-index discrimination observed in the ROC analysis, supporting the use of sCD40L and galectin-3 as key biomarkers for identifying high-risk patients with poor therapeutic response.

### Predictive efficacy of serum galectin-3 and sCD40L for treatment in AIS patients

**Figure 1:**
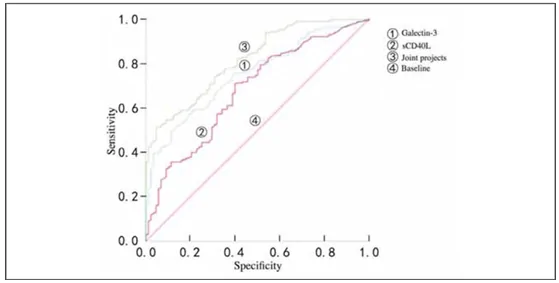
ROC curves of serum galectin-3 and sCD40L for ineffective treatment in AIS patients.

The ROC curve was plotted with the effective group as negative samples and the ineffective group as positive samples. Serum galectin-3 and sCD40L had respective area under the curve (AUC) values of 0.665 and 0.691 for forecasting therapy ineffectiveness in AIS patients, the specificities were 70.08% and 77.51%, respectively, and the sensitivities were 62.53% and 62.58%, respectively. The AUC predicted jointly by the two methods was 0.784 (Table 3 and Figure 1).

**Table 3:** Prediction efficacy of serum galectin-3 and sCD40L detection alone and in combination for ineffective treatment in AIS patients.

Indicator	AUC	AUC 95%CI	Best Truncation	Specificity (%)	Sensitivity (%)	P	Youden index
Galectin-3	0.665	0.536~0.795	10.232 ng/mL	70.08	62.53	<0.05	0.325
sCD40L	0.691	0.563~0.818	356.122 pg/L	77.51	62.58	<0.05	0.402
Two joint projects	0.784	0.678~0.889	-	65.18	81.25	<0.05	0.463

ROC curve analysis showed that the discriminative power of serum galectin-3 and sCD40L alone in predicting treatment ineffectiveness in AIS patients was moderate: The AUC of galectin-3 was 0.665 (sensitivity 62.53%, specificity 70.08%), and the AUC of sCD40L was 0.691 (sensitivity 62.58%, specificity 77.51%). After combining the two, the AUC rose to 0.784, the sensitivity increased to 81.25%, and the specificity was 65.18%, which was overall better than any single indicator. Combined detection can enhance the ability to identify individuals with poor therapeutic effects at a relatively high sensitivity, providing a more practical basis for early risk stratification and optimisation of treatment strategies.

### Serum levels of sCD40L and galectin-3 in AIS patients with various prognoses

The mRS score results revealed 38 patients in the poor-prognosis group and 142 in the good-prognosis group, with a poor-prognosis incidence rate of 21.11% (38/180). Compared to the group with a favourable prognosis, the group with an unfavourable prognosis had greater levels of serum galectin-3 and sCD40L (P<0.05) (Table 4).

**Table 4:** Serum galectin-3 and sCD40L levels in AIS patients with varying prognoses.

Group	n	Galectin-3 <br>(ng/mL)	sCD40L <br>(pg/L)
Poor prognosis <br>group	34	10.34±1.21	365.89±34.38
Good prognosis <br>group	146	7.55±1.94	266.18±50.21
t		3.544	13.823
P		<0.001	<0.001

After a 3-month follow-up, 142 cases had a good prognosis and 38 had a poor prognosis, for a poor-prognosis rate of 21.11%. A comparison of serological levels across different prognostic groups revealed that sCD40L and galectin-3 levels in the poor-prognosis group were significantly higher than those in the good-prognosis group (P<0.05), suggesting that elevated inflammation and fibrosis-related activities were associated with adverse outcomes. ROC analysis further confirmed its predictive value: the AUC for galectin-3 in predicting poor prognosis was 0.774 (sensitivity 75.06%, specificity 67.58%), and the AUC for sCD40L was 0.838 (sensitivity 80.04%, specificity 75.36%). The combined detection of the two achieved an AUC of 0.919, with a sensitivity of 90.63% and a specificity of 60.05%, which was improved compared with the single indicator, supporting its role as a key serum biomarker for prognostic stratification of AIS.

### Multivariate logistic regression investigation of the variables influencing AIS patients' poor prognosis

Serum galectin-3 and sCD40L levels in research participants following 4 weeks of treatment were utilised as independent variables (all input as original values), and the prognostic result of AIS patients (assigned values: good prognosis = 0; poor prognosis = 1) was used as the dependent variable. Multivariate logistic regression analysis was conducted. Elevated serum levels of sCD40L and galectin-3 were found to be risk factors for a poor outcome in patients with AIS (P<0.05) (Table 5).

**Table 5:** Multivariate logistic regression analysis of the influence of serum galectin-3 and sCD40L on the prognosis of AIS patients.

Factor	β	SE	Wald χ^2^	P	OR (95%CI)
Constant	-9.093	1.821	25.182	-	-
Galectinr-3	0.415	0.134	9.592	0.002	1.514 (1.165~1.969)
sCD40L	0.278	0.061	20.77	<0.001	1.320 (1.172~1.488)

After adjusting for confounding factors, elevated serum sCD40L and galectin-3 levels were independently associated with poor prognosis (all P<0.05), with positive correlations and stable effects. When the two indicators are included in the same model, they remain significant, suggesting that their biological information is complementary and can enhance the ability to identify adverse outcomes. This finding is consistent with ROC analysis results, supporting the use of sCD40L and galectin-3 as key serum biomarkers for three-month prognostic stratification and risk assessment in AIS.

### Predictive efficacy of serum galectin-3 ands CD40L for poor prognosis in AIS patients

**Figure 2:**
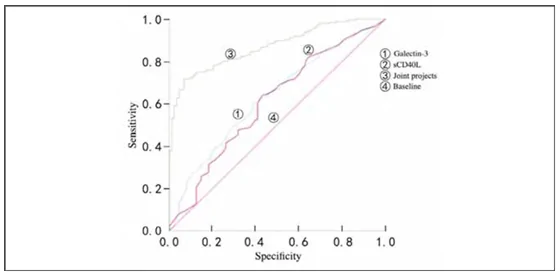
ROC curves of serum galectin-3 and sCD40L alone and in combination for predicting poor prognosis in AIS patients.

Positive samples from AIS patients with a bad prognosis and negative samples from AIS patients with a good prognosis were used to plot ROC curves. The results revealed that the AUCs for serum galectin-3 and sCD40L in predicting poor prognosis in AIS patients were 0.774 and 0.838, respectively, and the AUC for the combination of the two parameters was 0.919 (see Table 6 and Figure 2).

**Table 6:** Prediction efficacy of serum galectin-3 and sCD40L for poor prognosis in AIS patients.

Indicator	AUC	AUC 95%CI	Specificity <br>(%)	Sensitivity <br>(%)	P	Best Truncation <br>Value	Ybuden index
Galectin-3	0.774	0.665~0.884	67.58	75.06	<0.05	10.988 ng/mL	0.425
sCD40L	0.838	0.748~0.929	75.36	80.04	<0.05	356.343 pg/L	0.618
Two joint projects	0.919	0.860~0.978	60.05	90.63	<0.05	-	0.706

ROC curve analysis showed that serum galectin-3 and sCD40L had good discriminative power for the three-month prognosis of AIS patients: The AUC of galectin-3 was 0.774 (sensitivity 75.06%, specificity 67.58%), and that of sCD40L was 0.838 (sensitivity 80.04%, specificity 75.36%), among which sCD40L was superior to galectin-3. The AUC for combined detection of the two increased to 0.919; the sensitivity increased significantly to 90.63%, but the specificity decreased to 60.05%, suggesting that while the combined scheme improves the recognition rate for poor prognosis, it also yields more false positives. Overall, single indicators have moderate to good predictive efficacy. Combined detection further enhances the overall discrimination ability. In clinical applications, it is necessary to balance sensitivity and specificity and to optimise thresholds to achieve effective risk stratification.

## Discussion

The development of atherosclerosis can damage the inner lining of cerebral blood vessels, thereby accelerating arterial occlusion and stenosis, resulting in insufficient local brain perfusion, cerebral ischemia, and regional neurological dysfunction [20][21][22]. This is also the main pathological basis for AIS. Therefore, the main aim of clinical treatment for AIS patients is to improve cerebral blood perfusion and restore neurological function as much as possible [23]. Early intravenous thrombolysis is widely used, significantly improves cerebral blood flow recovery in AIS patients, and can effectively slow disease progression.

At present, imaging examinations still play an essential role in the clinical evaluation of the efficacy and prognosis of AIS patients [24]. However, this method is easily influenced by doctors' subjective judgment, and its ability to predict poor patient prognosis is relatively low. Therefore, it is crucial to identify more effective markers or examination measures to increase the accuracy of clinical diagnosis. Galectin-3 is a chimeric galectin with multiple biological activities. When ischemic injury occurs in brain tissue and neuronal cells are destroyed, galectin-3 is released and enters the bloodstream through the damaged blood-brain barrier. Studies [25][26][27] have shown that galectin-3 overexpression is closely associated with nerve cell damage and the number of diseased blood vessels and can serve as an effective marker for assessing the instability of atherosclerosis. Relevant studies [28][29][30] have shown that neurological function impairment in stroke patients is significantly correlated with serum galectin-3 levels (P<0.05). sCD40L is a soluble ligand derived from activated platelets that promotes blood coagulation and inflammatory responses. Patients with coronary atherosclerosis, carotid artery disease, and renal artery disease have markedly higher serum levels of sCD40L [31]. Both serum galectin-3 and sCD40L levels were associated with the therapeutic effect in AIS patients [32][33][34]. Serum galectin-3 and sCD40L alone had AUCs of 0.665 and 0.691 for predicting therapy ineffectiveness in AIS patients, respectively, and 0.774 and 0.838 for predicting poor prognosis, as determined by ROC analysis. These findings indicate that detecting serum galectin-3 and sCD40L levels can assist in the clinical assessment of treatment ineffectiveness and poor prognosis in patients at reasonable risk [35]. The AUCs for the combined detection of serum galectin and sCD40L in predicting ineffective treatment and poor prognosis in AIS patients increased to 0.784 and 0.919, respectively. Another study [36] reported that the prognosis of stroke patients and the level of sCD40L indicated that an excessively high level of sCD40L might be a risk factor for cerebral haemorrhage after treatment.

## Conclusion

The levels of serum sCD40L and galectin-3 in AIS patients with ineffective treatment and poor prognosis are significantly elevated. These factors contribute to the inadequate treatment and poor prognosis of AIS patients, helping clinical departments better evaluate treatment outcomes and prognosis.

## Dodatak

### Funding

None.

### Ethical approval

Our institution's Medical Ethics Committee approved the study, and each participant freely completed the informed consent form (HKYS-2025-A0211).

### Authors' contribution

Zhiqin Chen and Quanhua Xiao contributed equally to this work.

### Conflict of interest statement

All the authors declare that they have no conflict of interest in this work.
